# Are small additions solved by direct retrieval from memory or automated counting procedures? A rejoinder to Chen and Campbell (2018)

**DOI:** 10.3758/s13423-020-01818-4

**Published:** 2020-09-23

**Authors:** Catherine Thevenot, Pierre Barrouillet

**Affiliations:** 1 SSP, Institute of Psychology, University of Lausanne, Géopolis Building, Room 4536, CH-1015 Lausanne, Switzerland; 2 University of Geneva, Geneva, Switzerland

**Keywords:** Numerical cognition, Mental arithmetic, Strategies, Retrieval network, Automatization

## Abstract

Contrary to the longstanding and consensual hypothesis that adults mainly solve small single-digit additions by directly retrieving their answer from long-term memory, it has been recently argued that adults could solve small additions through fast automated counting procedures. In a recent article, Chen and Campbell (*Psychonomic Bulletin & Review*, *25*, 739–753, 2018) reviewed the main empirical evidence on which this alternative hypothesis is based, and concluded that there is no reason to jettison the retrieval hypothesis. In the present paper, we pinpoint the fact that Chen and Campbell reached some of their conclusions by excluding some of the problems that need to be considered for a proper argumentation against the automated counting procedure theory. We also explain why, contrary to Chen and Campbell’s assumption, the network interference model proposed by Campbell (*Mathematical Cognition, 1*, 121–164, 1995) cannot account for our data. Finally, we clarify a theoretical point of our model.

Scholars of numerical cognition generally agree that adults typically solve small single-digit additions (i.e., with a sum ≤ 10) by directly retrieving their answer from long-term memory (e.g., Ashcraft, 1982, 1992; Ashcraft & Guillaume, 2009; Campbell, 1995). It is assumed that the recurrent solving of these additions during childhood leads to the creation and strengthening of problem–answer associations in long-term memory to the point that the presentation of the problem would trigger the activation and retrieval of the associated answer (e.g., Siegler & Shrager, 1984). However, this consensus has recently been challenged by the alternative view that adults could solve small additions through fast and unconscious automated counting procedures involving the mental scanning of a portion of an ordered spatial or verbal representation such as a number line or a verbal number sequence (Barrouillet & Thevenot, 2013; Fayol & Thevenot, 2012; Mathieu, Gourjon, Couderc, Thevenot, & Prado, 2016; Mathieu et al., 2018; Thevenot, Barrouillet, Castel, & Uittenhove, 2016; Thevenot et al., 2020; Uittenhove, Thevenot, & Barrouillet, 2016). These procedures could be limited to very small addition problems involving operands from 1 to 4 because rapid mental scanning of a numerical sequence could be impossible beyond the size of the focus of attention estimated at about 4 items by Cowan (2001).

In a recent article, Chen and Campbell (2018) reviewed empirical findings and arguments on which the hypothesis of fast automated counting procedures is based and concluded that “the cumulative evidence for fast compacted procedures for adults’ simple addition does not justify revision of the long-standing assumption that direct memory retrieval is ultimately the most efficient process of simple addition for nonzero problems” (p. 740). However, there are three main problems with Chen and Campbell’s arguments.

First, Chen and Campbell (2018) contest the validity of the very small problem category with operands from 1 to 4 identified by Uittenhove et al. (2016), claiming that there is no boundary in RTs at *n* = 4 for *n* + 1 problems because there is a “very strong RT linearity for the *n* + 1 problems up to a sum of 8” (p. 743). Nevertheless, a simple look at the figure adapted from Uittenhove et al. (2016) and reported by Chen and Campbell (Fig. 2, p.743) reveals that it is not true. Solution times linearly increase between sums 3 and 5 (i.e., *N* from 2 to 4) and decrease at sum 6 (*N* = 5). Therefore, and as we claim, there is a boundary at *N* = 4. After *N* = 4, the pattern of solution times becomes very hectic with, as already stated, a decrease in solution times at *N* = 5, an increase at *N* = 6 and *N* = 7 (in a lesser extent), but a decrease again at *N* = 8 and an increase again at *N* = 9. The boundary at *N* = 4 for *n* + 1 problems is also revealed by the difference in problem size-related slopes between very small (28 ms) and medium (7 ms) problems. The boundary at *N* = 4 can be demonstrated using the same arguments for non-1 problems. The only way to reduce the difference in slopes between the two categories of problems is to ignore sum-to-9 and sum-to-10 problems, which Chen and Campbell repeatedly do, both for *n* + 1 problems and non-1 problems. Chen and Campbell justified not including problems with a sum of 10 because, “it has been observed for decades that they are fast and accurate relative to their magnitude” (p. 743). They justified not including the sum-to-9 problems because they “could benefit from proximity to the very high memory strength sum-to-10 problems” (p. 744). However, there was no evidence in Uittenhove et al. (2016) for the special status of sum-to-10 problems, which were not solved faster than sum-to-9 problems in *n* + 1 or non-1 problems. Moreover, the mechanism by which memory traces of sum-to-9 problems would benefit from proximity to highly available traces is not described by Chen and Campbell, and we cannot see by which cognitive theory it could be accounted for. Moreover, if sum-to-9 problems benefit from the proximity of sum-to-10 problems, sum-to-11 problems should benefit from the same advantage. However, it was not at all the case, as Uittenhove et al.’s adult participants took almost 3 seconds more on average to solve sum-to-11 than sum-to-10 problems (Fig. 1 in Uittenhove et al., 2016, p. 294). Finally, it has to be noted that the absence of solution times variations for medium problems was not specific to sum-to-10 and sum-to 9 problems, but was also observed for sum-to-8 problems, at least when the whole population tested by Uittenhove et al. (2016) was considered (again, see their Fig. 1). There was, indeed, no significant difference in solution times between sum-to-8 and sum-to-9 problems, *t* = 1.44, *p* = .15.

Second, Chen and Campbell (2018) claim that the problem-size effect in very small additions (with operands from 1 to 4) can be predicted by the network interference model proposed by Campbell (1995), which assumes that the problem size on RTs is due to interference occurring during the retrieval of arithmetic facts. However, the network interference model also predicts comparable size effects for the other small problems. Nevertheless, as already stated, Uittenhove et al. (2016) observed that outside the range of very small additions, problems with a sum from 7 to 10 do not present a size effect at all (mean slope of −5 ms), even when sum-to-10 problems are removed from the analysis (mean slope of 1 ms). Thus, Chen and Campbell (2018) model can only account for a subset of Uittenhove et al. (2016) results, but is contradicted by the overall RTs pattern.

Finally and on a more theoretical level, Chen and Campbell (2018) argue against the automated counting procedure theory because “there is no evidence they are aware of that the Arabic digits up to four automatically activate a corresponding number of tokens in spatial working memory, but the Arabic digits for five or higher do not” (p. 744). In fact, Uittenhove et al. (2016) never said that Arabic digits up to 4 automatically activate their analogical representation, while larger numbers do not. What is automatic is the run of the sequence of steps when the goal to solve the problem has been formulated and the encoding of each operand in a single focus of attention is possible. Thus, contrary to what Chen and Campbell allude, it is not because operands larger than 4 do not automatically activate a representation in spatial working memory that they are not processed by the automated procedure. This is because their analogical representation, which is required by the procedure to run, cannot be held within a single focus of attention.
